# 3,5,3′-Triiodo-L-Thyronine- and 3,5-Diiodo-L-Thyronine- Affected Metabolic Pathways in Liver of LDL Receptor Deficient Mice

**DOI:** 10.3389/fphys.2016.00545

**Published:** 2016-11-17

**Authors:** Maria Moreno, Elena Silvestri, Maria Coppola, Ira J. Goldberg, Li-Shin Huang, Anna M. Salzano, Fulvio D'Angelo, Joel R. Ehrenkranz, Fernando Goglia

**Affiliations:** ^1^Department of Science and Technologies, University of SannioBenevento, Italy; ^2^Department of Medicine, Columbia UniversityNew York, NY, USA; ^3^Proteomics and Mass Spectrometry Laboratory, Instituto per Il Sistema Produzione Animale in Ambiente Mediterraneo, National Research CouncilNapoli, Italy; ^4^Biogem s.c.a r.l., Ariano Irpino (AV)Italy; ^5^Department of Medicine, Intermountain HealthcareSalt Lake City, UT, USA

**Keywords:** iodothyronines, liver, cholesterol, LDL receptor, proteomics

## Abstract

3,5,3′-triiodo-L-thyronine (T3) and 3,5-diiodo-L-thyronine (T2), when administered to a model of familial hypercholesterolemia, i.e., low density lipoprotein receptor (LDLr)-knockout (*Ldlr*^−/−^*)* mice fed with a Western type diet (WTD), dramatically reduce circulating total and very low-density lipoprotein/LDL cholesterol with decreased liver apolipoprotein B (ApoB) production. The aim of the study was to highlight putative molecular mechanisms to manage cholesterol levels in the absence of LDLr. A comprehensive comparative profiling of changes in expression of soluble proteins in livers from *Ldlr*^−/−^ mice treated with either T3 or T2 was performed. From a total proteome of 450 liver proteins, 25 identified proteins were affected by both T2 and T3, 18 only by T3 and 9 only by T2. Using *in silico* analyses, an overlap was observed with 11/14 pathways common to both iodothyronines, with T2 and T3 preferentially altering sub-networks centered around hepatocyte nuclear factor 4 α (HNF4α) and peroxisome proliferator-activated receptor α (PPARα), respectively. Both T2 and T3 administration significantly reduced nuclear HNF4α protein content, while T2, but not T3, decreased the expression levels of the HNFα transcriptional coactivator PGC-1α. Lower PPARα levels were found only following T3 treatment while both T3 and T2 lowered liver X receptor α (LXRα) nuclear content. Overall, this study, although it was not meant to investigate the use of T2 and T3 as a therapeutic agent, provides novel insights into the regulation of hepatic metabolic pathways involved in T3- and T2-driven cholesterol reduction in *Ldlr*^−/−^ mice.

## Introduction

Familial hypercholesterolemia (FH) is a common inherited disorder resulting from mutations in the low-density lipoprotein receptor (LDLr) gene leading to defects in LDL cholesterol (LDL-C) clearance. Despite the use of currently available cholesterol-lowering treatments, a high proportion of FH patients do not reach treatment goals and remain at risk of atherosclerotic cardiovascular diseases (Naoumova et al., 2004). More intensive and alternative treatments are often needed for these patients as well as other hypercholesterolemic patients who do not meet treatment goals or who have difficulty with statin therapy. One possible approach to cholesterol reduction is via activation of hepatic thyroid receptors (TRs).

Both 3,5,3′,5′-tetraiodo-L-thyronine (T4), and 3,5,3′-triiodo-L-thyronine (T3) (THs) reduce circulating cholesterol in animals and humans (Klein and Danzi, 2008; Angelin and Rudling, 2010). However, their therapeutic use for the treatment of hyperlipidemia is limited due to deleterious side effects from TR activation in extrahepatic tissues leading to altered cardiovascular function, muscle wasting, and bone loss (Scheiffele and Schultze, 1972). In recent years, several thyromimetics (i.e., GC1 and KB2115) have been developed that selectively activate TRβ, the predominant TR isoform in the liver that is primarily responsible for the effects on cholesterol and lipoprotein metabolism exerted by T3 (Johansson et al., 2005; Erion et al., 2007; Tancevski et al., 2010; Pramfalk et al., 2011). These compounds retard atherosclerosis progression in animals and exert favorable lipid-modulating effects in humans, while lacking THs-related thyrotoxic side-effects (Bakker et al., 1998; Erion et al., 2007; Baxter and Webb, 2009; Pedrelli et al., 2010). However, side effects may limit its clinical use since the cholesterol-reducing thyrometic eprotirome (Ladenson et al., 2010) was recently shown to elevate circulating liver enzymes (Sjouke et al., 2014), a sign of liver damage and, indeed, development of eprotirome was terminated after cartilage damage was observed in a toxicological study in dogs.

Hepatic TR activation might lower cholesterol levels via multiple mechanisms. Although the primary hepatic effects on cholesterol metabolism of THs was thought to be via LDL clearance through increased expression of LDLr (Ness and Lopez, 1995), we and others have reported that iodothyronines [T3 and 3,5-diiodo-L-thyronine (T2)] as well as GC1 markedly reduce circulating LDL-C in LDLr knockout (*Ldlr*^−/−^) mice (Goldberg et al., 2012; Lin et al., 2012). These studies and those of others (Davidson et al., 1988, 1990) show that both iodothyronines lead to a reduction in hepatic secretion of apolipoprotein B (ApoB)-containing lipoproteins [very low density lipoproteins (VLDL) and LDL] while high density lipoprotein (HDL) levels are reduced only by T3 (Goldberg et al., 2012).

This reduction is not associated with increased hepatic protein or mRNA levels of LDL receptor related protein (LRP) 1 or the scavenger receptor-B1 (SR-B1) (Goldberg et al., 2012). Cholesterol reduction was also reported to be associated with increased expression of cholesterol-7 α-hydroxylase (Cyp7a1) (Lin et al., 2012), which converts cholesterol into bile acids. These studies (Goldberg et al., 2012; Lin et al., 2012) dispelled the conventional view that thyroid-mediated reduction of cholesterol requires LDLr and indicated that the thyroid hormone responsive element (TRE) in the LDL receptor is not required *in vivo* for thyroid-induced LDL reduction. Although our previous study (Goldberg et al., 2012) with high doses of T3 and T2 was not meant to investigate their use as therapeutic agents, the elicited dramatic reduction in circulating cholesterol levels in hypercholesterolemic *Ldlr*^−/−^ mice opened new perspectives in defining non-LDLr pathways that may have potential for the treatment of hypercholesterolemia.

The goal of the following study was to gain additional insights into the molecular factors and pathways that contribute to the above hypocholesterolemic actions of T2 and T3. To uncover the pathways and networks altered by these two iodothyronines, we performed a comprehensive comparative profiling of changes in expression of soluble proteins in livers from *Ldlr*^−/−^ mice treated with either T3 or T2.

## Materials and methods

### Animals and experimental protocol

All studies were approved by the Columbia University Institutional Animal Care and Use Committee (IACUC). Male wild-type (WT) C57BL/6 and *Ldlr*^−/−^ mice of 3–4 months of age, purchased from the Jackson Laboratory, were used. WT C57BL/6 and *Ldlr*^−/−^ mice (*n* = 5–6/group) were fed a Western-type diet (WTD) containing: 42% fat, 42.7% carbohydrate, 15.2% protein, 0.15% cholesterol; total 4.5 Kcal/g (Harlan Teklad) for 1 week. After 1 week, C57BL/6 and *Ldlr*^−/−^ mice were continued on the WTD and were divided into groups receiving vehicle (58.5% saline + 40% DMSO + 1.5% 1M NaOH) or iodothyronines—T3 (0.75 mg/kg) (EMD Chemicals/Calbiochem) or T2 (12.5 mg/kg) (Santa Cruz)—via daily gavage for another week and then sacrificed. The doses of T3 and T2 were chosen to obtain the same cholesterol reducing effect (Goldberg et al., 2012). Mass spectrometry and NMR profiles showed no T3 or T4 contamination in the T2. All blood samples obtained during the non-terminal portion of the study were taken after a 4-h fast.

### Protein extraction and sample preparation for two-dimensional gel electrophoresis (2D-E)

Protein extraction and sample preparation for 2D-E were performed as reported in Silvestri et al. (2006). Liver tissue was suspended in sample buffer [20 mM Tris, 7 M urea, 2 M thiourea, 4% CHAPS, 10 mM 1,4-dithioerythritol (DTE), 1 mM EDTA, and a mixture of protease and phosphatase inhibitors]. The suspensions were homogenized using a Polytron homogenizer, sonicated for 30 s, and centrifuged at 150,000 g for 45 min. The obtained supernatants contained the total liver proteins solubilized in the isoelectrofocusing (IEF)-compatible agents. The protein content of each sample was determined by Bio-Rad's DC method (Bio-Rad Laboratories, Hercules, CA). Total protein extracts were prepared for each animal, and each individual was assessed separately.

### 2D-E

Samples of 650 μg of protein were applied to immobilized pH gradient (IPG) strips (pH 4–7, 17 cm) (Bio-Rad). Samples of 1 mg of protein were utilized for preparative gels (IPG strips, pH 4–7, 17 cm). Focusing started at 200 V, with the voltage being gradually increased to 3500 V and kept constant for a further 66,500 V/h (PROTEAN IEF System, Bio-Rad). Prior to SDS-PAGE, the IPG strips were incubated for 15 min with a solution of Tris-HCl buffer (pH 8.8), urea (6 M), glycerol (30%, v/v), SDS (2%, w/v), and DTT (2%, w/v). Strips were then equilibrated for another 15 min in the same buffer containing iodoacetamide (2.5%, w/v) instead of DTT. The second-dimensional separation was performed in 12% SDS-polyacrylamide gels. After protein fixation, the gels were stained with colloidal Coomassie Blue, according to the manufacturer's instructions. Molecular masses were determined by running standard protein markers, covering the range 10–200 kDa. The pI values used were those given by the supplier of the IPG strips.

### Protein visualization and image analysis

Digital images of the gels were recorded using a calibrated densitometer (GS-800, Bio-Rad) and analyzed using PDQuest software (Bio-Rad) (Silvestri et al., 2006). For each matchset analysis, maps corresponding to protein extracts from animals of the same experimental group were organized into “Replicate Groups” (each containing 4 maps), named *Ldlr*^−/−^, *Ldlr*^−/−^+T2, and *Ldlr*^−/−^+T3. Statistical analysis was performed using a Student's *t*-test. Spots for which the *p*-value was <0.05 were considered to display significant changes.

### Protein digestion and mass spectrometry analysis

Spots from 2D-E were manually excised from gels, triturated, and washed with water. Proteins were in-gel reduced, S-alkylated, and digested with trypsin, as previously reported (D'Ambrosio et al., 2008). Protein digests were subjected to a desalting/concentration step on μZipTipC18 pipette tips (Millipore Corp., Bedford, MA, USA) and then analyzed by nano-liquid chromatography (nLC)-electrospray ionization (ESI)-linear ion trap (LIT)-tandem (MS/MS) mass spectrometry, using a LTQ XL mass spectrometer (Thermo Fischer Scientific, USA) equipped with a Proxeon nanospray source connected to an Easy-nanoLC (Proxeon, Odense, Denmark). Peptide mixtures were separated on an Easy C18 column (100 × 0.075 mm, 3 μm) (Thermo, USA) using a gradient of acetonitrile containing 0.1% formic acid in aqueous 0.1% formic acid; acetonitrile was ramped from 5 to 35% over 10 min, from 35 to 95% over 2 min, and remained at 95% for 12 min, at a flow rate of 300 nL/min. Spectra were acquired in the range m/z 400–2000. Acquisition was controlled by a data-dependent product ion-scanning procedure over the 3 most abundant ions, enabling dynamic exclusion (repeat count 2; exclusion duration 1 min). The mass isolation window and collision energy were set to m/z 3 and 35%, respectively.

### Protein identification

MASCOT software package version 2.2.06 (Matrix Science, UK) (Cottrell, 2011) was used to identify spots unambiguously from a *Mus Musculus* protein sequence database retrieved from UniProt repository (76,058 sequences, 10/2011). Raw data from nanoLC-ESI-LIT-MS/MS were searched using a mass tolerance value of 2 Da for precursor ion and 0.8 Da for MS/MS fragments, trypsin as proteolytic enzyme, a missed-cleavages maximum value of 2, and Cys carbamidomethylation and Met oxidation as fixed and variable modifications, respectively. Protein candidates with more than 2 assigned peptide sequences, with MS/MS ion score >30 and a peptide expectation value <0.05, were further evaluated by comparison with their calculated mass and pI values, using the experimental values obtained from 2D-E.

### *In silico* biological analysis

The lists of differentially expressed proteins were input into the IPA platform (Ingenuity Systems, http://www.ingenuity.com) for the identification of canonical pathways and functions differing between the treatments. The cutoff used was 1.5 for the fold change and 0.05 for the *p*-value. Fisher's exact test was used to calculate a *p*-value indicating the probability that each biological function and/or disease assigned to that dataset might be so assigned due to chance alone. The results of Fisher's exact test were corrected for multiple testing using the false discovery rate (FDR). Comparison analysis was also performed to compare the roles of the proteins among the generated lists. In order to generate the reported networks, a list of differentially expressed proteins in the experimental conditions was overlaid onto a global molecular network developed from information contained in the Ingenuity Pathways Knowledge Base (IPKB). Networks of these focus-gene products were then algorithmically generated on the basis of their connectivity. The IPA platform uses a curated database to construct functional regulatory networks from a list of individual proteins. To build networks, the program utilizes the IPKB containing large numbers of individually modeled relationships between proteins (obtained from the upgraded literature contained in IPA platform). The algorithm then determines a statistical score for each network. This is done by comparing the number of focus proteins that contribute to a given network relative to the total number of occurrences of those proteins in all networks or pathways stored in the IPKB. Then a score is assigned to each network. The score is the negative log of P, and it denotes the likelihood that the focus proteins in the network might be found together by chance. Therefore, scores of 2 have at least 99% confidence of not being generated by chance alone. In addition, the biological functions assigned to each network are ranked according to the significance of that biological function to the network.

### Nuclei preparation and western immunoblot analysis

As already described in Cioffi et al. (2010), livers were dissected and minced in ice-cold isolation buffer (consisting of 220 mM mannitol, 70 mM sucrose, 20 mM Tris-HCl, 1 mM EDTA, and 5 mM EGTA, pH 7.4) and then were homogenized in a Potter-Elvehjem homogenizer. To isolate nuclei, the liver homogenate was centrifuged at 500 g for 10 min at 4°C. The obtained pellet was subsequently resuspended and spun through a sucrose cushion (30% sucrose, 10 mM Tris-HCl, pH 7.5, 10 mM NaCl, and 3 mM MgCl_2_) at 1300 g for 10 min at 4°C. The nuclear pellet was washed with cold 10 mM Tris-HCl, pH 7.5, and 10 mM NaCl.

For Western immunoblotting analysis, the nuclear pellets were homogenized in lysis buffer containing 20 mM Tris-HCl, pH 7.5, 150 mM NaCl, 1 mM EDTA, 1 mM EGTA, 2.5 mM Na_2_H_2_P_2_O_7_, 1 mM b-CH_3_H_7_O_6_PNa_2_, 1 mM Na_3_VO_4_, 1 mM PMSF, 1 mg/ml leupeptin, and 1% (w/v) Triton X-100 by using an Optima TLX Ultraturrax (Beckman Coulter, Milan, Italy), then centrifuged at 13,400 g for 10 min at 4°C. Protein concentration was determined by using the Bio Rad's DC method (Bio-Rad Laboratories, Hercules, CA). The following primary antibodies were used: anti-HNF4α (ab41,898, Abcam, mouse monoclonal); anti-PPARα (ab8934, Abcam, rabbit polyclonal); anti-LXRα (ab41,902, Abcam, mouse monoclonal); anti-TRβ (GTX113278, GeneTex, rabbit polyclonal); anti-PGC-1α (AB3242, Merck Millipore, rabbit polyclonal); anti-α tubulin (ab4074, Abcam, rabbit polyclonal). Proteins were detected by a chemiluminescence protein-detection method based on the protocol supplied with a commercially available kit (Millipore) and by using the appropriate secondary antibodies. Signals were quantified by means of a Bio-Rad ChemiDoc™ XRS, using dedicated software (QuantityOne, Bio-Rad Laboratories).

### Serum levels of free T3 (FT3) and free T4 (FT4)

The serum levels of FT3 and FT4 were measured by commercially available kits by means of immunoassay [materials and protocols supplied by Byk-Sangtec Diagnostica (Dietzenbach, Germany)].

### Statistical analysis

Data are expressed as the mean ± SD as indicated in the figures. Comparisons between two groups were performed using student's *t*-test. Comparisons amongst three groups were performed using one-way ANOVA and followed by the Newman-Keuls test. For all analyses, *p* < 0.05 was considered statistically significant.

## Results

### Altered liver protein expression profile induced by T2 and T3

**Figure 1:**
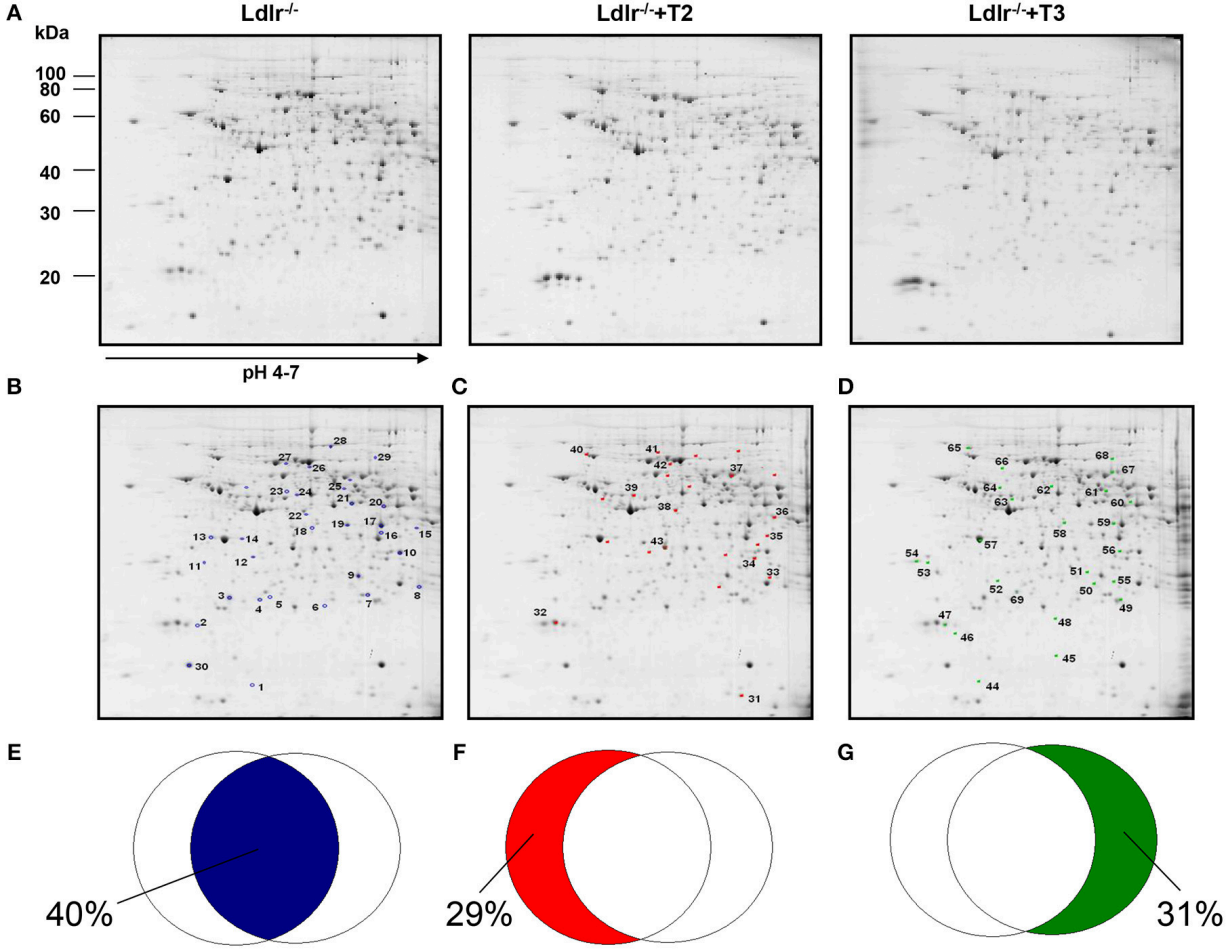
**Effects of T3 and T2 on the hepatic proteome in *Ldlr*^−/−^ mice. (A)** Representative 2D-E Coomassie Blue stained maps of total liver soluble proteins extracted from *Ldlr*^−/−^, *Ldlr*^−/−^+T2, and *Ldlr*^−/−^+T3 mice and separated on 17 cm/pH 4–7 IPG strips in the first dimension and on 12% SDS-PAGE in the second. Spotted and identified proteins among those with a density that differed significantly (by at least 0.5- or 2-fold; *p* < 0.05) between *Ldlr*^−/−^ and either *Ldlr*^−/−^+T2 or *Ldlr*^−/−^+T3 (blue spots) **(B)**, *Ldlr*^−/−^ and *Ldlr*^−/−^+T2 only (red spots) **(C)**, and *Ldlr*^−/−^ and *Ldlr*^−/−^+T3 only (green spots) **(D)**. Overlapped circles represent the total amount of differentially expressed proteins between the experimental groups. The blue area represents the percentage of proteins affected by both T2 and T3 (iodothyronines' common proteomic effect in liver of *Ldlr*^−/−^ mice) **(E)**; the red area represents the percentage of proteins affected only by T2 treatment **(F)**; the green area represents the percentage of proteins affected only by T3 treatment **(G)**.

Through a 2D-E-based proteomic approach, the hepatic pathways and the molecular mediators involved in the T2- and T3- induced reductions in circulating cholesterol levels in *Ldlr*^−/−^ mice were investigated. At the detection-limits set, the image software counted 450 matched proteins among the various maps in the liver (Figure 1A). Pair-wise comparisons were performed to analyze the differential expression pattern associated with the T2 and T3 treatment (*Ldlr*^−/−^+T2 and *Ldlr*^−/−^+T3 vs. *Ldlr*^−/−^). When the interest was limited to a differential expression of at least 2-fold and a statistical significance of at least 95% (*p* < 0.05), 57 (about 12.4% of total entries), and 59 spots (about 12.8% of total entries) showed significant quantitative changes in liver following T2- and T3-treatment, respectively. Importantly, the differential expression produced by T2 and T3 overlapped on 33 protein products (Figure 1B) corresponding to 40% of the total amount of differentially expressed proteins (Figure 1E). The remaining, specifically affected either by T2 (Figure 1C) or by T3 (Figure 1D), represented 29 and 31% of the total amount of differentially expressed proteins (Figures 1F,G, respectively).

**Figure 2:**
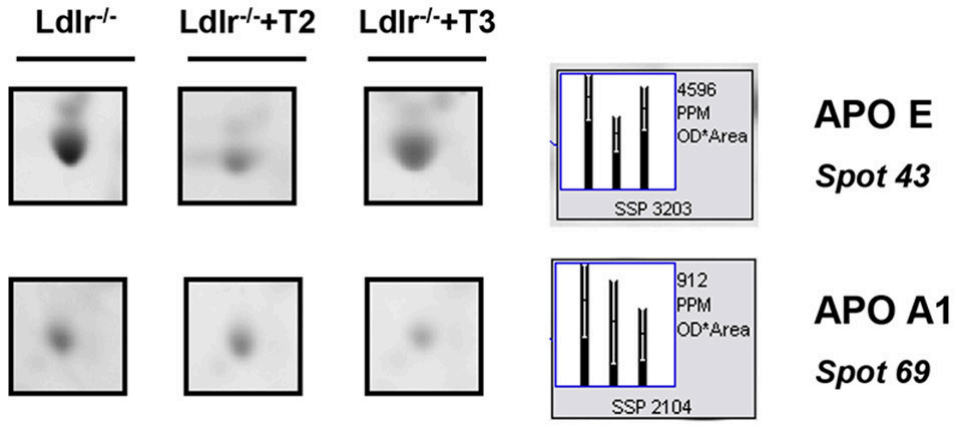
**Effects of T3 and T2 on the intrahepatic content of ApoE and ApoA1 in *Ldlr*^−/−^ mice**. Representative subsections of 2D-E images of livers of *Ldlr*^−/−^, *Ldlr*^−/−^+T2, and *Ldlr*^−/−^+T3 mice are reported. Results are expressed as arbitrary units (means ± SD; *n* = 4).

Considering that in a previous study (Goldberg et al., 2012) we demonstrated a modulation exerted by T3 and T2 on intrahepatic content of apolipoproteins and that ApoE and ApoA1 are well resolved on a standard 2D map of total soluble liver proteome, ApoE and ApoA1 were localized by means of map comparison (Fountoulakis et al., 2001) and subjected to quantitative analysis. ApoE, corresponding to spot 43 (Figure 1C), was significantly affected only by T2 with a differential expression of −40% vs. *Ldlr*^−/−^ control levels (*p* < 0.1) (Figure 2). ApoA1, corresponding to spot 69 (Figure 1D), tended to be reduced in *Ldlr*^−/−^+T3 mice by −40% vs. *Ldlr*^−/−^ control levels, although without statistical significance (Figure 2). These two proteins together with other 67 spots among those automatically detected by the matching software, were manually excised, trypsinyzed, and subjected to nanoLC-ESI-LIT-MS/MS analysis. Fifty-two protein spots were unambiguously identified (Supplementary Material 1). Among these, 25 protein products were affected by both T2 and T3, 9 only by T2, 18 only by T3.

Proteins detected as a mixture of components were excluded from further analyses and thus were not discussed further in this report. When proteins were identified as multiple spots on the same map, putatively reflecting the occurrence of post-translational modifications, the pattern of changes was fairly similar among the various species.

### Analysis of potential target proteins of T2 and T3

**Figure 3:**
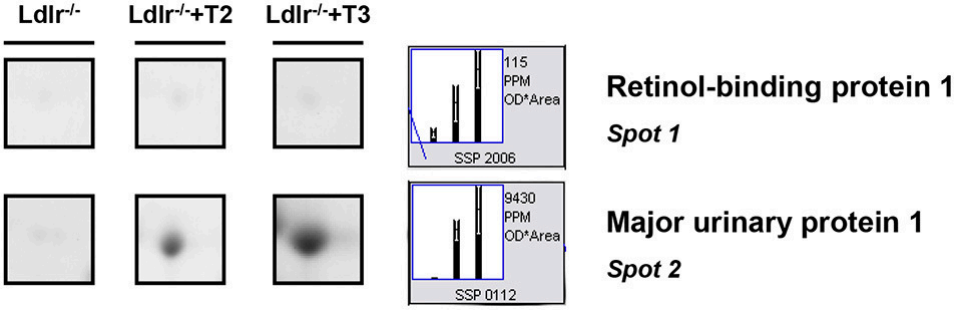
**T3- and T2-dependent differential expression in liver of *Ldlr*^−/−^ mice: increased proteins**. Representative subsections of 2D-E images of livers of *Ldlr*^−/−^, *Ldlr*^−/−^+T2, and *Ldlr*^−/−^+T3 mice. Results are expressed as arbitrary units (means ± SD; *n* = 4) (*p* < 0.05).

**Figure 4:**
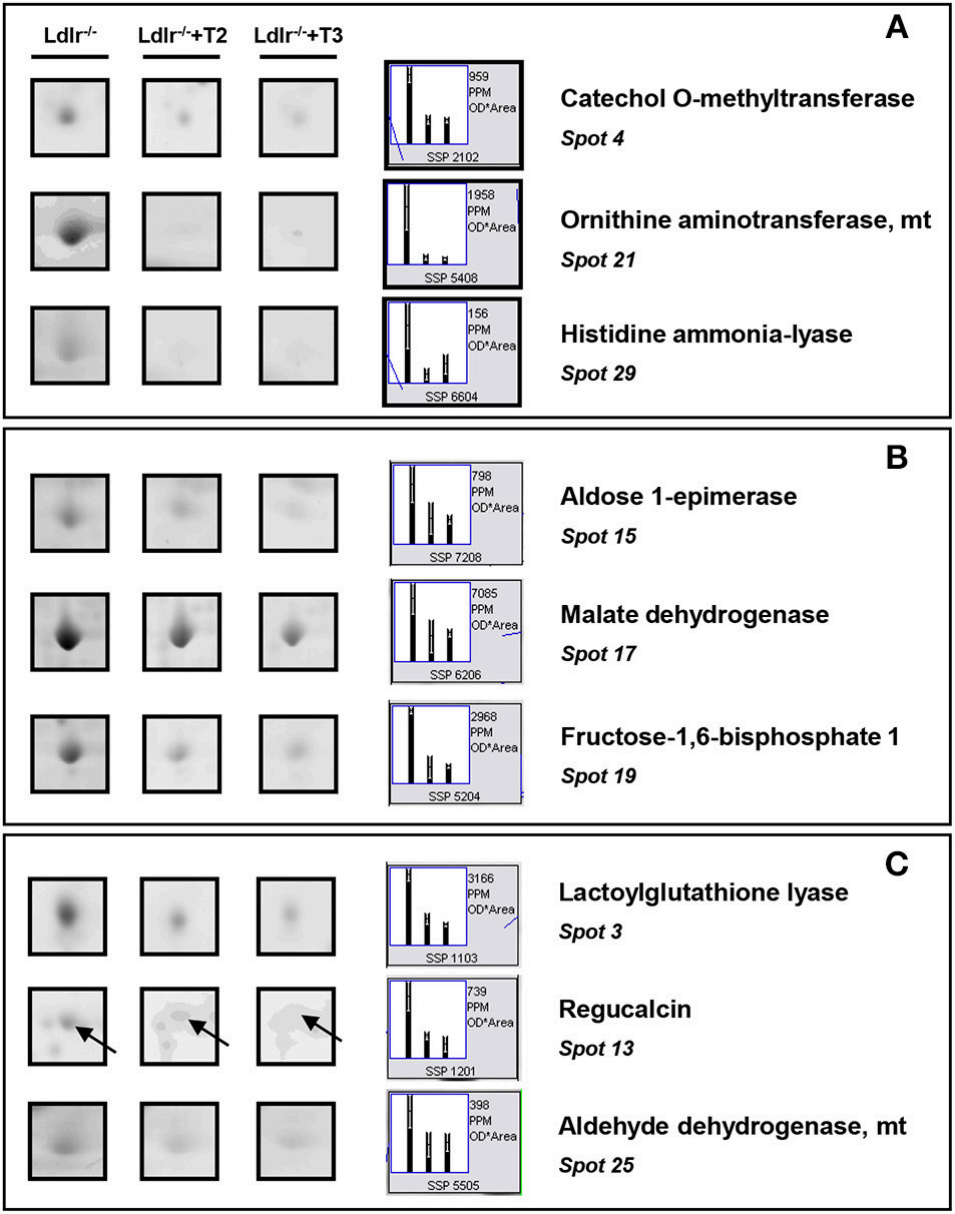
**T3- and T2-dependent differential expression in liver of *Ldlr*^−/−^ mice: decreased proteins**. Representative subsections of 2D-E images of livers of *Ldlr*^−/−^, *Ldlr*^−/−^+T2, and *Ldlr*^−/−^+T3 mice. **(A)** Enzymes involved in amino acid metabolism. **(B)** Enzymes involved in substrate metabolism. **(C)** Proteins involved in cellular stress. Results are expressed as arbitrary units (means ± SD; *n* = 4) (*p* < 0.05).

Both T2 and T3 reduced expression of a number of hepatic proteins compared to the expression levels obtained for *Ldlr*^−/−^ control mice (91 and 79% of differentially expressed proteins, respectively). A significant increase produced by both iodothyronines was observed for cellular retinol-binding protein 1 also named CRBP1 (spot 1) and major urinary protein 1 (MUP1) (spot 2), with the effect of T3 being more pronounced than that of T2 (Figure 3). Key metabolic enzymes were among the proteins decreased by both iodothyronines (Figure 4). Importantly, these enzymes are involved in major hepatic processes such as amino acid metabolism [catechol O-methyltransferase (spot 4), ornithine aminotransferase, mitochondrial (spot 21) and histidine ammonia-lyase (spot 29)] (Figure 4A), substrate metabolism [aldose 1-epimerase (spot 15), malate dehydrogenase (spot 17), and fructose-1,6-bisphosphatase 1 (spot 19)] (Figure 4B), and cellular stress [lactoylglutathione lyase (spot 3), regucalcin (spot 13), mitochondrial aldehyde dehydrogenase (spot 25)] (Figure 4C). Of note, these proteins are regulated to the same extent by either T3 or T2, highlighting a common effect of the two iodothyronines.

**Figure 5:**
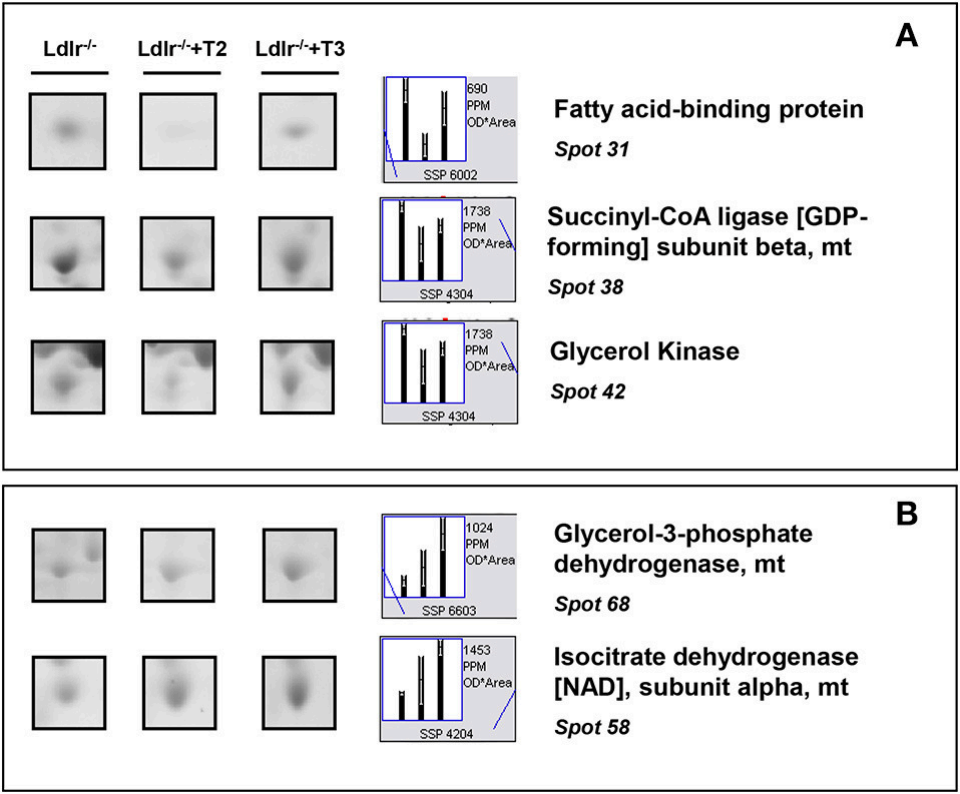
**Iodothyronine specific effects on differential expression in liver of *Ldlr*^−/−^ mice. (A)** Representative T2 specific affected proteins. **(B)** Representative T3 specific affected proteins. Results are expressed as arbitrary units (means ± SD; *n* = 4) (*p* < 0.05).

T2 specifically decreased proteins such as fatty acid-binding protein (spot 31), succinyl-CoA ligase (spot 38), and glycerol kinase (spot 42), which are involved in lipid, oxidative, and carbohydrate metabolism, respectively (Figure 5A). In contrast, glycerol-3-phosphate dehydrogenase (spot 68) and isocitrate dehydrogenase [NAD] (spot 58), involved in oxidative and substrate metabolism, were significantly increased only by T3 (Figure 5B). As a whole, these results suggest that, at the doses used, T2 and T3, although modulating overlapping metabolic events (e.g., amino acid and intra-mitochondrial energy metabolism), also have their own specific molecular targets.

### IPA analysis of potential target proteins involved in T2 and T3 effects

**Figure 6:**
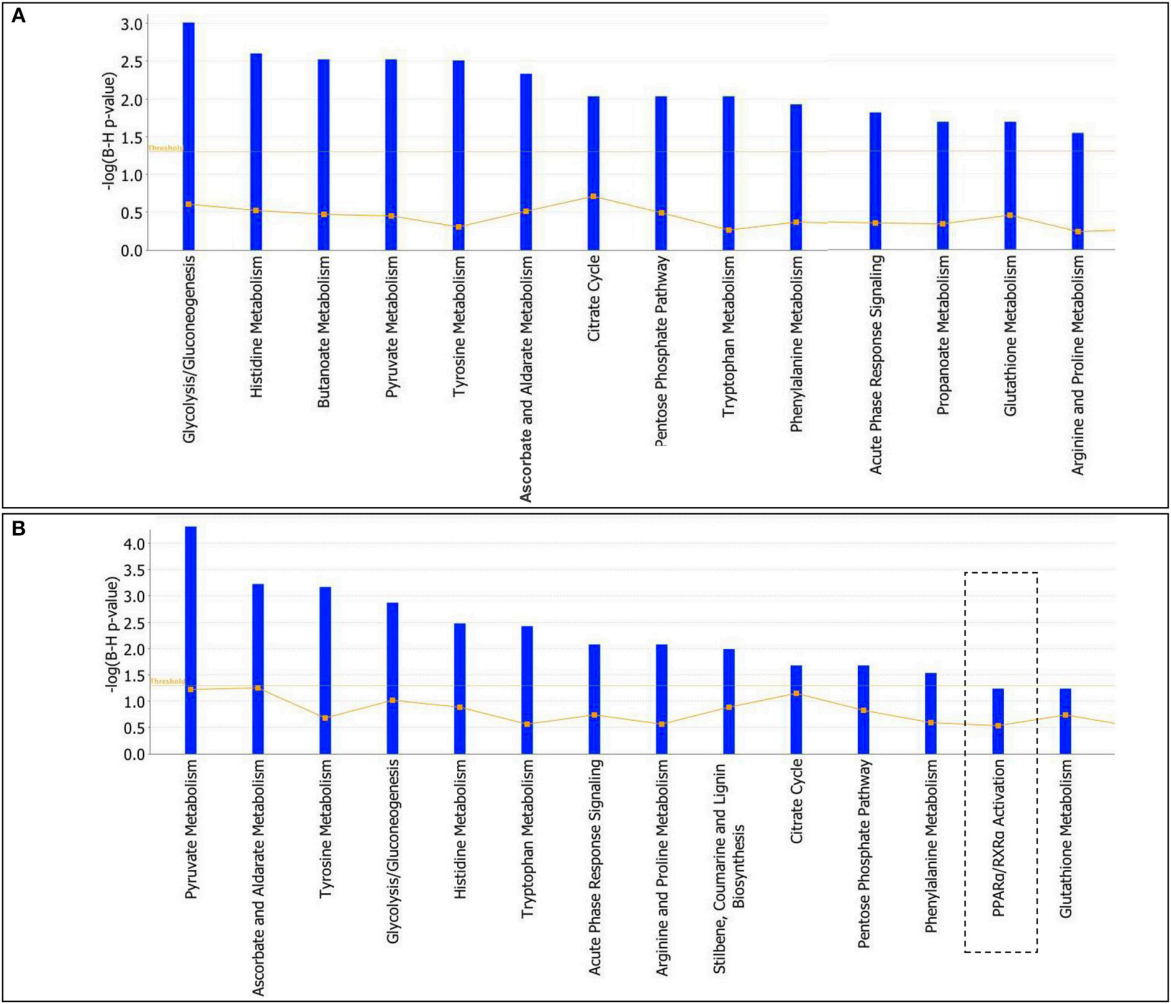
**T3 and T2 affected canonical pathways in liver of *Ldlr*^−/−^ mice: *in silico* analysis**. The lists of differentially expressed proteins were input into the IPA platform (Ingenuity® Systems, http://www.ingenuity.com) for functional enrichment analysis. Over-represented canonical pathways for *Ldlr*^−/−^+T2 **(A)** and *Ldlr*^−/−^+T3 **(B)** vs. *Ldlr*^−/−^ deregulated proteins are represented in barplots by their statistical score (negative logarithm to the base 10 of B-H corrected *p*-value) with a threshold of 1.3 (yellow straight line). The ratio between deregulated and all proteins of a pathway is also reported (yellow not straight line).

To further characterize the effects elicited by either T2- or T3-treatment in liver of *Ldlr*^−/−^ mice, proteomic data were analyzed by using the IPA platform that, based on known interactions between affected proteins, defines common functional and canonical pathways as well as protein networks, thereby offering additional information about the complex interactive links between modulated proteins following the treatments under study. The *in silico* analysis confirmed that the most significant T2/T3- dependent changes altered lipid-, amino acid-, carbohydrate-, and energy- metabolism (Supplementary Material 2). These changes are mediated by effects on pathways such as glycolysis/gluconeogenesis, citrate cycle, pentose phosphate, glutathione, and amino acid metabolism (Figures 6A,B). Overall, in terms of modulated functions and pathways, T2 and T3 exerted a similar effect with a few exceptions. Of particular metabolic relevance the peroxisome proliferator-activated receptor α (PPARα)/retinoid X receptor α (RXRα) pathway was affected only by T3 (Figure 6B).

**Figure 7:**
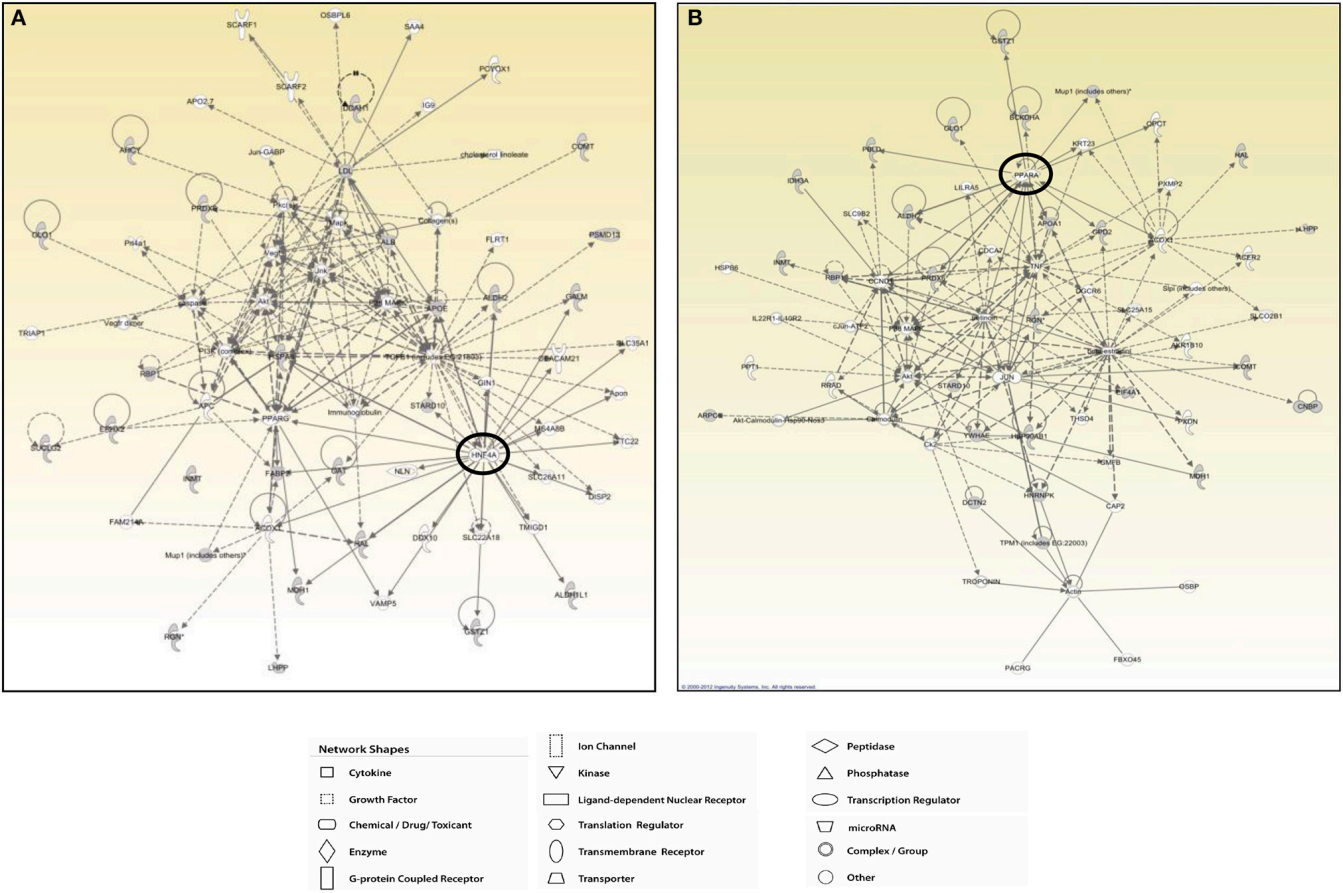
**T3 and T2 affected protein networks in liver of *Ldlr*^−/−^ mice**. Network representation of the molecular relationships between identified, differentially expressed proteins (Ingenuity Systems Ltd.) in liver of *Ldlr*^−/−^+T2 **(A)** and *Ldlr*^−/−^+T3 **(B)** vs. *Ldlr*^−/−^ mice. Gene products are represented as nodes, and the biological relationship between two nodes is represented as an edge (line). Indirect interactions appear as broken lines, whereas direct interactions appear as solid lines. All edges shown are supported by at least 1 reference from the literature, from a textbook, or from canonical information stored in the Ingenuity Pathways Knowledge Base. Human, mouse, and rat orthologues of a gene are stored as separate objects in IPKB, but are represented as a single node in the network. For clarity, network shapes are shown.

The protein network analysis for T2 produced the highest scored node (the value being 28) corresponding to the hepatocyte nuclear factor 4α (HNF4α), a nuclear receptor well known to act as a master regulator of liver-specific gene expression orchestrating lipid and cholesterol metabolism (Figure 7A). HNF4α is directly interconnected with some focus proteins acquired in 2D-E analysis such as aldehyde dehydrogenase (ALDH2, ALDH1), malate dehydrogenase (MDH1), and fatty acid-binding protein (FABP), which are involved in substrate and lipid metabolism (Figure 7A). Strictly in line with the pathways analysis, as far as T3 effects are concerned, network tool revealed PPARα as the main hub of the highest scored network (IPA score 30), a nuclear receptor that regulates diverse aspects of lipid metabolism, including fatty acid oxidation, and lipoprotein metabolism (Figure 7B). Within the network, PPARα directly interacts with some focus proteins acquired in 2D-E analysis such as aldehyde dehydrogenase (ALDH2), mitochondrial glycerol-3-phosphate dehydrogenase (GPD2), maleylacetoacetate isomerase (GSTZ1), major urinary protein 1 (MUP1), and peroxiredoxin-6 (PRDX6), all of which are involved in substrate and energy metabolism and in cellular stress (Figure 7B).

### Nuclear factors affected by T2 and T3 in liver of *Ldlr^−/−^* mice

**Figure 8:**
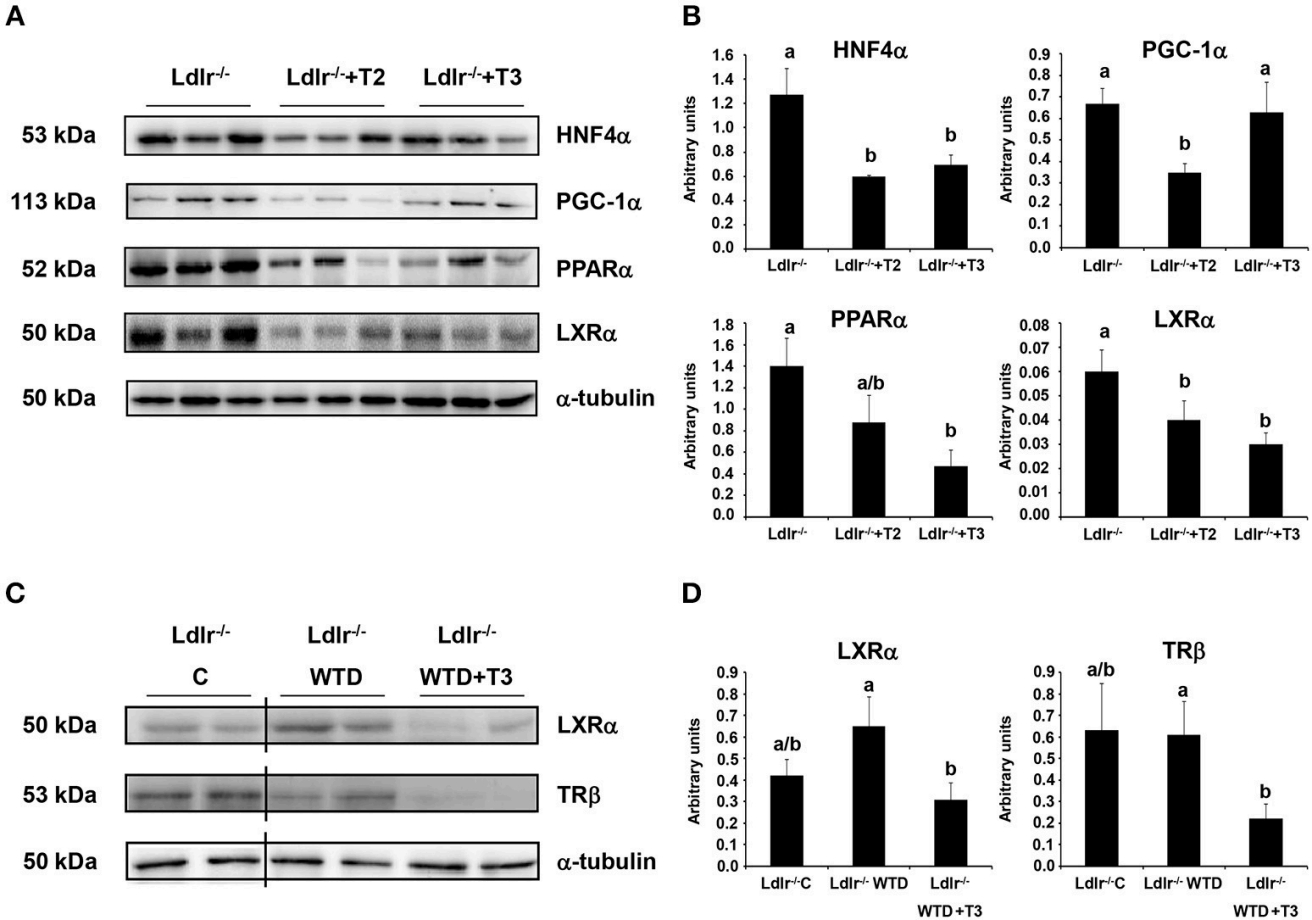
**Effects of T3 and T2 on nuclear factors in liver of *Ldlr*^−/−^ mice. (A)** Representative Western blot analyses of HNF4α, PGC-1α, PPARα, and LXRα protein levels in *Ldlr*^−/−^, *Ldlr*^−/−^+T2, and *Ldlr*^−/−^+T3 mice. Liver nuclear protein contents were normalized on α-tubulin. Protein load was 30 μg/lane. **(B)** Quantitative analysis of protein expression levels. Results are expressed as arbitrary units (means ± SD; *n* = 3). Bars labeled with dissimilar letters are significantly different (*p* < 0.05). **(C)** Representative Western blot analyses of LXRα and TRβ protein levels in *Ldlr*^−/−^ mice fed with either a chow (*Ldlr*^−/−^ C) or a western type diet (*Ldlr*^−/−^ WTD) and treated with T3 (*Ldlr*^−/−^ WTD+T3). Liver nuclear protein contents were normalized on α-tubulin. Protein load was 30 μg/lane. The vertical lines indicate cuts in the membranes to avoid to display samples not related to the used model. **(D)** Quantitative analysis of nuclear protein content of LXRα and TRβ in *Ldlr*^−/−^ C, *Ldlr*^−/−^ WTD and *Ldlr*^−/−^ WTD+T3 mice. Results are expressed as arbitrary units (means ± SD; *n* = 4). Bars labeled with dissimilar letters are significantly different (*p* < 0.05).

Considering that IPA network analysis highlighted HNF4α and PPARα as the highest scored nodes grouping the major number of interactions with differentially regulated proteins by T2 and T3, respectively, Western blot analysis was performed to further investigate the putative involvement of such nuclear factors into the hypocholesterolemic effects of both iodothyronines. Both T2 and T3 administration to *Ldlr*^−/−^ mice produced a significant reduction of the nuclear content of HNF4α, with the effect being slightly stronger in *Ldlr*^−/−^+T2 than in *Ldlr*^−/−^+T3 mice (Figures 8A,B). By contrast, only T3 administration produced a pronounced and significant decrease of nuclear PPARα levels vs. those in control *Ldlr*^−/−^ mice. According to what is suggested by the *in silico* analysis, this result is consistent with the hypothesis that PPARα has a specific role in the metabolic effects exerted by T3 in *Ldlr*^−/−^ mice.

In view of the fact that a partnership of HNF4α with its coactivator, peroxisome proliferator-activated receptor γ coactivator-1α (PGC-1α) in the regulation of lipoprotein metabolism has been characterized (Rhee et al., 2006), the effect of T2 and T3 on the hepatic nuclear content of PGC-1α was analyzed. As shown in Figures 8A,B, only T2 significantly reduced expression levels of PGC-1α, hence suggesting an impaired PGC-1α/HNF4α-dependent signaling in the plasma cholesterol-lowering effects of T2.

Nuclear receptors other than HNF4α and PPARα also participate in the transcriptional regulation of key factors involved in the intrahepatic cholesterol metabolism. Specifically, it has been reported that T3 exerts its hypocholesterolemic effect mainly by binding to liver TRβ but, in the absence of this, also acting through the liver X receptor α (LXRα) (Gullberg et al., 2000). Nuclear receptor expression levels may be predictive for the activity of their ligands. In view of this and considering that LXRα is a central hepatic cholesterol sensor, Western blot analysis was performed to verify whether the hypocholesterolemic effect of T2 and T3 in *Ldlr*^−/−^ mice correlates with LXRα and TRβ hepatic expression levels. In parallel with the lower plasma cholesterol levels detected in *Ldlr*^−/−^+T2 and *Ldlr*^−/−^+T3 mice (vs. *Ldlr*^−/−^), both iodothyronines reduced LXRα nuclear content (Figures 8A,B). Of note, LXRα nuclear content tended to increase when *Ldlr*^−/−^ mice were consuming the WTD (Figures 8C,D). A significant reduction of TRβ levels (about −60% vs. *Ldlr*^−/−^ control mice) was observed only in *Ldlr*^−/−^+T3 mice (Figures 8C,D).

### Nuclear factors affected by T2 and T3 in livers of WT mice

In order to evaluate whether the absence/presence of LDLr might be a major determinant of the effects of T2 and T3 on the expression level of the aforementioned transcription factors, Western blot analyses were performed on liver tissue from WT mice fed either chow diet or WTD. The effects elicited by T2 on HNF4α, PGC-1α, and LXRα levels in livers from *Ldlr*^−/−^ mice were not observed in WT mice fed chow or WTD while T3 treatment, without significantly altering PPARα nuclear content, increased both HFN4α and PGC-1α hepatic levels, in WTD WT mice (Supplementary Material 3).

Finally, due to the large dose of T2 used, we sought to exclude displacement of T3 from thyroid binding globulin as a reason for the efficacy of the T2-treatment. To do this, we assessed serum FT3 and FT4 levels in T2 treated mice; neither was increased [the actual values being for FT3 (pg/ml): 8.55 ± 1.8; 3.2 ± 1.9^*^; for FT4 (ng/dl): 1.24 ± 0.8; 0.15 ± 0.06^*^, in WTD WT and WTD-WT+T2 animals, respectively; ^*^P < 0.05 vs. WTD WT]. Therefore, T2 did not displace T3 from the thyroid binding globulin. Thus, increased endogenous T3 does not account for the effects of T2.

## Discussion

Although it has been hypothesized on the basis of *in vitro* studies that THs mediate lowering of LDL-C primarily by increased hepatic expression of LDLr (Lopez et al., 2007) and that they up regulate scavenger SR-B1 in mice (Johansson et al., 2005), very recently, we (Goldberg et al., 2012) and others (Lin et al., 2012) reported that THs can reduce serum cholesterol via a LDLr-independent mechanism. *Ldlr*^−/−^ mice have modestly elevated levels of plasma cholesterol but when fed a WTD they develop much higher levels of cholesterol and atherosclerosis. When treated with high doses of T3 or T2, WTD-fed *Ldlr*^−/−^ mice showed a dramatic decrease in LDL-C with these reductions being linked to reductions in ApoB48 and ApoB100 secretion (Goldberg et al., 2012).

Utilizing 2D-E and MS, we studied a total proteome of 450 liver proteins and identified 25 proteins affected by both T2 and T3, 18 only by T3 and 9 only by T2. At the used doses, both T2 and T3 impact liver proteome producing a general decrease of modulated proteins.

When the differentially expressed proteins were mapped for the identification of canonical pathways and biological functions (among which lipid, carbohydrate and amino acid metabolism), 14 pathways/lists with the highest statistical significance were identified for both iodothyronines. By comparing these lists, 3 non-overlapping pathways were identified for one of the two iodothyronines (e.g., of particular metabolic relevance, PPARα/RXRα activation for T3). This indicates the existence of common proteomic targets for T2 and T3 as well as of specific ones.

Of note, among the individual common proteins increased by both iodothyronines, we identified MUP1. Although the physiological functions of this low molecular weight secreted protein belonging to the lipocalin family remain poorly understood, recent evidence demonstrated that MUP1 could be positively correlated with energy expenditure, metabolism, and insulin sensitivity in mice (Hui et al., 2009). This might open new perspectives in understanding the mechanisms through which T3 and T2 determine the whole animal metabolic state (for recent review see, Mullur et al., 2014; Goglia, 2015; Davis et al., 2016).

Key enzymes involved in amino acid- and substrate- metabolism and cellular stress were all individual common proteins decreased by both iodothyronines. Specifically, the reduction of catechol O-methyltransferase, ornithine aminotransferase, and histidine ammonia-lyase indicates a suppressive effect elicited by both T2 and T3 on enzymes involved in amino acid degradation and ureagenesis, which is in line with the known effects of thyroid hormone on ammonia metabolism (Sochor et al., 1981; Marti et al., 1988). The reduction in the expression levels of lactoylglutathione lyase and aldehyde dehydrogenase, on the other hand, indicates a lower content of detoxifying enzymes in the liver of *Ldlr*^−/−^ mice treated with either T2 or T3. Of note, consistent with what was previously reported (Silvestri et al., 2006), we observed an iodothyronine-induced decrease of the hepatic level of regucalcin, suggesting a role of calcium availability in the cellular effects of T2 and T3 (Del Viscovo et al., 2012).

Only T3 increased the mitochondrial enzymes glycerol-3-phosphate dehydrogenase and isocitrate dehydrogenase. This suggests that T3, in *Ldlr*^−/−^ mice, might stimulate mitochondrial respiration and substrate oxidation. In particular, glycerol-3-phosphate dehydrogenase is a well-established marker of liver thyroid status (Rauchová et al., 2011).

Analyzing the functional interaction between the identified differentially expressed target proteins, the network tool revealed sub networks centered around HNF4α for T2 and around PPARα for T3.

HFN4α, an orphan member of the nuclear receptor superfamily, is crucial for the maintenance and regulation of hepatic lipid metabolism and it is a major *in vivo* regulator of the expression of liver-specific genes involved in the production and clearance of apolipoproteins (Yin et al., 2011). Odom et al. (2004) reported that HFN4α directly binds to almost 50% of the actively transcribed hepatic genes whereas no other transcription factor binds to more than 1/5 of that number. In humans, Medica 16, an HFN4α antagonist, acts as a hypolipidemic agent likely due to reduced activation of HFN4α target gene expression (Hertz et al., 2001; Sheena et al., 2005; Rufibach et al., 2006). The identification of HFN4α as a central node in the proteomic effects of T2 suggests that this receptor could be involved in the hypocholesterolemic effect elicited by T2 in *Ldlr*^−/−^ mice. Indeed, accordingly with the dramatic reduction in circulating apoB-containing lipoproteins, nuclear HFN4α protein content was strongly decreased in liver from WTD-*Ldlr*^−/−^ mice following T2 treatment.

Interacting proteins are crucial in determining the transcriptional activity of nuclear receptors. The PGC-1α/HFN4α partnership plays a crucial role in hepatic lipoprotein metabolism (lipoprotein synthesis and export) (Rhee et al., 2006), and modulation of PGC-1α coactivation of HFN4α may provide a novel mechanism to manage dyslipidemia (Rhee et al., 2006). Nuclear hepatic PGC-1α content was reduced by T2, thus suggesting affected PGC-1α- HFN4α-dependent signaling in the plasma cholesterol-lowering effects of T2. This might be correlated with the decreased hepatic level of ApoE as well as the decreased plasma levels of ApoB48 and ApoB100 and decreased *Srb1* mRNA levels (Goldberg et al., 2012). When compared with T2, T3 decreased HFN4α nuclear content while not affecting that of PGC-1α, likely indicating that the observed T2/T3-hypocholesterolemic effect depend on different molecular events. Besides PGC-1α, another coactivator of HFN4α is FABP, which has been shown to structurally and functionally bind HNF4α to stimulate its transcriptional activity (McIntosh et al., 2013). The significantly lower level of FABP in liver from WTD-*Ldlr*^−/−^ mice only following T2 treatment is in line with the decreased expression of HNF4α and its protein targets. Among these, of a particular interest could be the glycerol kinase (GK), an enzyme involved in supplying glucogenic precursors for hepatic glucose production. The GK promoter contains a functional HNF4α binding site (Stepanian et al., 2003) and the hepatic content of this enzyme was significantly decreased only in T2-treated mice. By contrast, T3 treatment, while decreasing liver HNF4α levels, did not decrease those of FABP. Moreover, the lack of a significant effect of T3 on GK liver content might suggest a differential impact of the two iodothyronines on hepatic glucose production and thus on the whole animal glycaemia. This would require further analyses above all considering the well-known insulin antagonizing effect of T3 and the recently described insulin sensitizing action of T2 (de Lange et al., 2011; Moreno et al., 2011; Padron et al., 2014).

Reduction of PPARα levels was found only following T3 treatment and it is in accordance with the lower levels of HDL and *Srb1* mRNA as well as the increased hepatic triglyceride secretion (Goldberg et al., 2012). Although PPARα activation by fibrates decreases ApoB-containing lipoproteins as well as total serum ApoB levels in rodents and human (Peters et al., 1997; Milosavljevic et al., 2001), treatment of *Ldlr*^−/−^ mice with ciprofibrate markedly decreased plasma ApoB-48-carrying IDL and LDL but at the same time caused a marked accumulation of ApoB-100 carrying IDL/LDL, increased plasma cholesterol levels and promoted aortic atherosclerosis (Fu et al., 2004). Our finding of a T3-induced decrease in PPARα levels associated with a significant reduction in plasma cholesterol and ApoB-containing lipoproteins levels as well decreased hepatic production of ApoB-100, is in line with the above study and supports the cholesterol-lowering effect of T3 in a situation in which the IDL/LDL lipoprotein remnants cannot be efficiently cleared from the plasma via the LDLr pathway. In the context of our results, it is interesting to note that PPARα is a target of HNF4α (Pineda Torra et al., 2002; Martinez-Jimenez et al., 2010) and that T3 by reducing HNF4α levels likely produces a downstream negative effect on PPARα which, indeed, could not be the case with T2, that did not significantly reduce PPARα levels vs. controls. So although both T2 and T3 reduce HNF4α levels, likely they target different intrahepatic pathways all leading to the hypocholesterolemic effect in *Ldlr*^−/−^ mice.

Whether specifically targeting HNF4α- and PPARα-downstream proteins will reproduce the LDL reduction found with thyroid hormones is an area for future studies also based on *in vitro* approaches. Moreover, we should note that 2D-E based proteomic analyses are intrinsically limited as they resolve only soluble and highly expressed components of protein mixtures, and as the detection of low and high molecular mass or of basic and hydrophobic proteins is inefficient. Thus, other factors missing in our analysis could also be critical for the hypocholesterolemic effects of iodothyronines.

Besides the above mentioned nuclear receptors, LXRα, by regulating expression of several genes (Calkin and Tontonoz, 2010), controls cholesterol clearance via regulation of intestinal cholesterol absorption, biliary cholesterol secretion and cholesterol conversion into bile acids. Both T3 and T2 lowered nuclear LXRα protein content. One competitor receptor of LXRα in mediating the hypocholesterolemic effect of T3, is TRβ (Gullberg et al., 2000). According to the hyperthyroid state of the animals, hepatic nuclear content of TRβ was significantly decreased only in T3-treated mice. The lack of T2 effects on TRβ levels, at the high dose of T2 used, considering the low, but not ignorable, affinity of T2 for TRβ, might stimulate to further investigate how T2 might exert some of its transcriptomic/proteomic effects in a TR-dependent manner (Mendoza et al., 2013; Navarrete-Ramírez et al., 2014; Orozco et al., 2014; Jonas et al., 2015), above all in view of the fact that, as already shown by Goldberg et al. (2012) in *Ldlr*^−/−^ mice, also in WT mice, the high used dose of T2 significantly suppressed FT3 and FT4 levels. Moreover, as far as it concerns the differences between the actions exerted by T3 and T2, it has to be considered that only one dose of each iodothyronine was used and T2 was administered at a much higher concentration than T3, so that it would be expected that differences in response, apart from involving different mechanisms, might also reflect differences in the dose/response curve.

T2-mediated effects on HNF4α, PGC-1α, and LXRα levels were not observed in WT mice either when fed with Chow or WTD. On the other hand, T3 lead to increased HFN4α and PGC-1α levels in WT WTD fed mice. Thus, the absence/presence of LDLr may be a major determinant of the effects that T2 and T3 exert on the above nuclear factors.

In conclusion, although there are differences among mice, humans and rats regarding cholesterol hepatic metabolism, this study, independently of the specific effects elicited by the high used doses of T2 and T3, furnishes novel information on LDLr-independent pathways and mediators that could be important targets for cholesterol lowering therapies.

## Author contributions

MM designed the experimental approaches, supervised data elaboration, wrote and revised the manuscript; ES designed the experimental approaches, performed proteomic analyses, supervised data elaboration, wrote and revised the manuscript; MC performed proteomic analyses, elaborated data, wrote and revised the manuscript; IG designed the experimental model and approaches, supervised animal care and treatments, and revised the manuscript; LH designed the experimental model and approaches, supervised animal care and treatments and revised the manuscript; AS performed MS analyses and revised the manuscript; FD performed *in silico* analyses and revised the manuscript; JE contributed to the design of the work and revised the manuscript; FG coordinated the experimental procedures and revised the manuscript.

## Funding

University of Sannio Research Grants.

### Conflict of interest statement

The authors declare that the research was conducted in the absence of any commercial or financial relationships that could be construed as a potential conflict of interest.
