# Supplementary material for: 3,5,3′-Triiodo-L-Thyronine- and 3,5-Diiodo-L-Thyronine- Affected Metabolic Pathways in Liver of LDL Receptor Deficient Mice
Source: Front Physiol. 2016 Nov 17;7:545. doi: 10.3389/fphys.2016.00545 (PMC5112267; doi:10.3389/fphys.2016.00545)
Supplement: Supplementary file 1 [file Table1.pdf]

Supplementary Material 1. List of unambiguously identified proteins

SPOT	NAME	EMPAI [1]	gene name	Swiss prot Acc Numb	theoretical MW (Da)	pI	Protein Score	Peptides	coverage
1	Retinol-binding protein 1	1,97	Rbp1	Q00915	15714,92	5,11	288	6	40
2	Major urinary protein 1	79,45	Mup1	P11588	18694,86	4,84	929	19	75
3	Lactoylglutathione lyase	5,34	Glo1	Q9CPU0	20678,41	5,25	635	13	57
4	Catechol O-methyltransferase	2,73	Comt	O88587	29496,37	5,52	466	9	36
6	Ferritin light chain 1	1,75	Ftl1	P29391	20671,2	5,65	354	6	39
7	Uncharacterized protein	2,5	Abhd14b	E9QN99	22450,77	6,04	442	9	46
8	Maleylacetoacetate isomerase	4,7	Gstz1	Q9WVL0	24275,22	7,68	624	11	66
9	Indolethylamine N-methyltransferase	2,68	Inmt	P40936	29459,8	6,00	572	9	29
10	3-hydroxyanthranilate 3,4-dioxygenase	3,56	Haao	Q78JT3	32804,3	6,10	803	15	59
11	Phospholysine phosphohistidine inorganic pyrophosphate phosphatase	1,97	Lhpp	Q9D7I5	29144,3	4,98	367	8	44
12	Phenazine biosynthesis-like domain-containing protein 2	2,39	Pbld2	Q9CXN7	31983,36	5,19	585	11	40
13	Regucalcin	5,68	Rgn	Q64374	33406,85	5,16	776	17	62
14	Regucalcin	10,33	Rgn	Q64374	33406,85	5,16	940	19	69
15	Aldose 1-epimerase	0,33	Galm	Q8K157	37798,61	6,26	171	3	9
16	PCTP-like protein	1,63	Stard10	Q9JMD3	32951,46	6,67	485	7	29
17	Malate dehydrogenase, cytoplasmic	1,19	Mdh1	P14152	36379,97	6,16	366	7	24
18	N(G),N(G)-dimethylarginine dimethylaminohydrolase 1	2,44	Ddah1	Q9CWS0	31249,78	5,64	462	11	45
19	Fructose-1,6-bisphosphatase 1	4,12	Fbp1	Q9QXD6	36781,28	6,18	1025	18	50
20	Adenosylhomocysteinase	3,13	Ahcy	P50247	47688,16	6,08	1133	18	50
21	Ornithine aminotransferase, mitochondrial	8,17	Oat	P29758	45790,52	5,73	1314	28	64
22	Uncharacterized protein	2,51	Psmd13	E9Q5I9	39694,77	5,14	626	15	42
25	Aldehyde dehydrogenase, mitochondrial	0,56	Aldh2	P47738	54374,87	6,05	303	7	18
28	Aldehyde dehydrogenase family 1 member L1	1,68	Aldh1l1	Q8R0Y6	98709,17	5,64	1462	28	34
29	Histidine ammonia-lyase	1,7	Hal	P35492	72257,88	5,94	990	21	38
30	Cytochrome b5	6,81	Cyb5a	P56395	15109,8	4,94	528	10	55
31	Fatty acid-binding protein, intestinal	2,16	Fabp2	P55050	14994,99	6,87	236	5	37
32	Major urinary protein 6	55,23	Mup6	P02762	18695,8	4,72	1041	27	81
33	Peroxiredoxin-6	2,11	Prdx6	O08709	24739,45	5,72	496	8	52
37	Putative uncharacterized protein	13,78	Ephx2	Q3UQ71	62543,33	5,96	1854	54	66
38	Succinyl-CoA ligase [GDP-forming] subunit beta, mitochondrial	5,76	Suclg2	Q9Z2I8	42695,06	5,42	1008	32	46
40	78 kDa glucose-regulated protein	1,34	Hspa5	P20029	70464,55	5,01	1094	18	38

**41**	Serum albumin	1,93	Alb	P07724	65892,1	5,53	957	23	35
**42**	Glycerol kinase	0,2	Gk	Q64516	61227,45	5,62	198	3	7
**43**	Apolipoprotein E	8,86	Apoe	P08226	33968,15	5,46	1051	18	50
**45**	Actin-related protein 2/3 complex subunit 5 OS	3,46	Arpc5	ARPC5	16335	5,47	386	7	49
**46**	Major urinary protein 4	1,75	Mup2	A2AKN9	20920	4,94	306	6	37,2
**47**	Major urinary protein 5	94,24	Mup8	A2AKN8	20878	4,89	826	13	60,6
**48**	Cellular nucleic acid binding protein	1,77	Cnbp	Q3ULK8	20704	8	288	6	33,9
**50**	Uncharacterized protein	2,48	Prdx6	D3Z0Y2	22594	8,97	443	8	38,5
**51**	Endoplasmic reticulum resident protein 29	2,43	Erp29	ERP29	28862	5,9	430	7	27,1
**53**	Uncharacterized protein	8,13	Tpm1	E9Q455	28959	4,77	1017	20	62,2
**54**	Tyrosine 3-monooxygenase/tryptophan 5-monooxygenase activation protein	78,34	Ywhae	Q5SS40	29326	4,63	1243	21	62,4
**57**	Regucalcin	240,89	Rgn	RGN	33899	5,15	1620	25	64,9
**58**	Isocitrate dehydrogenase [NAD] subunit alpha, mitochondrial	4,99	Idh3a	IDH3A	40069	6,27	743	14	33,9
**60**	Branched chain ketoacid dehydrogenase E1	4,52	Bckdha	Q99L69	50612	8,15	986	16	49,3
**61**	Uncharacterized protein	2,47	Ldhd	E9Q5A1	52164	5,71	1096	15	35,3
**63**	Eif4a1 protein (Fragment)	4,13	Eif4a1	Q4FZL1	46222	5,32	750	12	37,5
**64**	Dynactin subunit 2	2,12	Dctn2	DCTN2	44204	5,14	830	14	42
**65**	Heat shock protein 84b	1,28	Hsp90ab1	Q71LX8	83571	30,2	884	18	30,2
**66**	Heterogeneous nuclear ribonucleoprotein K	1,61	Hnrnpk	B2M1R6	48760	5,38	736	14	38
**68**	Glycerol-3-phosphate dehydrogenase, mitochondrial	2,8	Gpd2	GPDM	81416	6,17	1696	28	39,5
**69**	Putative uncharacterized protein	1,26	Apoa1	Q3V2G1	30684,62	5,64	470	9	36

1 Exponentially Modified Protein Abundance Index (emPAI) as calculated by MS results.
