# Supplementary figures and images for: 3,5,3′-Triiodo-L-Thyronine- and 3,5-Diiodo-L-Thyronine- Affected Metabolic Pathways in Liver of LDL Receptor Deficient Mice
Source: Front Physiol. 2016 Nov 17;7:545. doi: 10.3389/fphys.2016.00545 (PMC5112267; doi:10.3389/fphys.2016.00545)

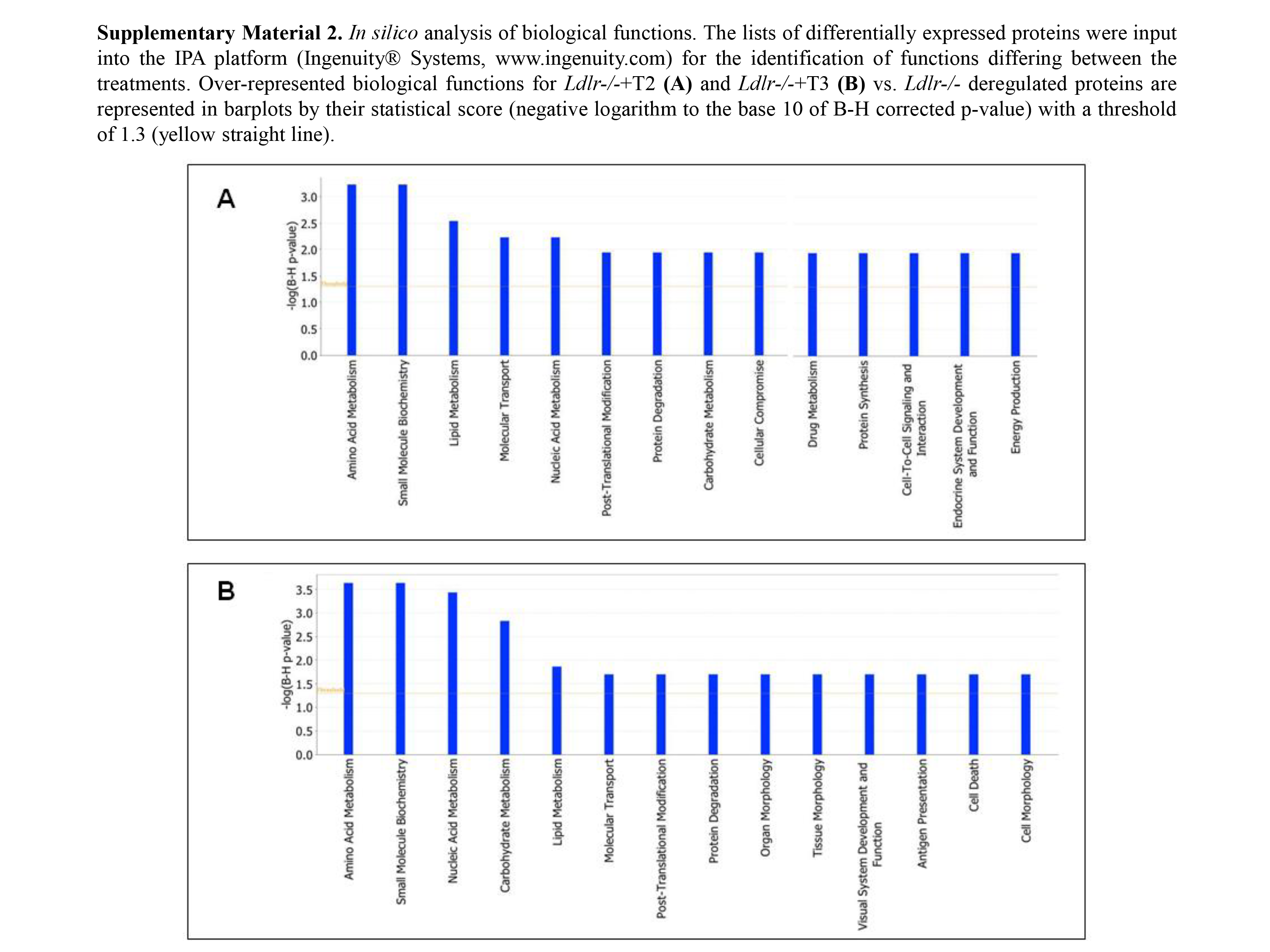

Supplement: Supplementary file 2 [file Image1.tif]

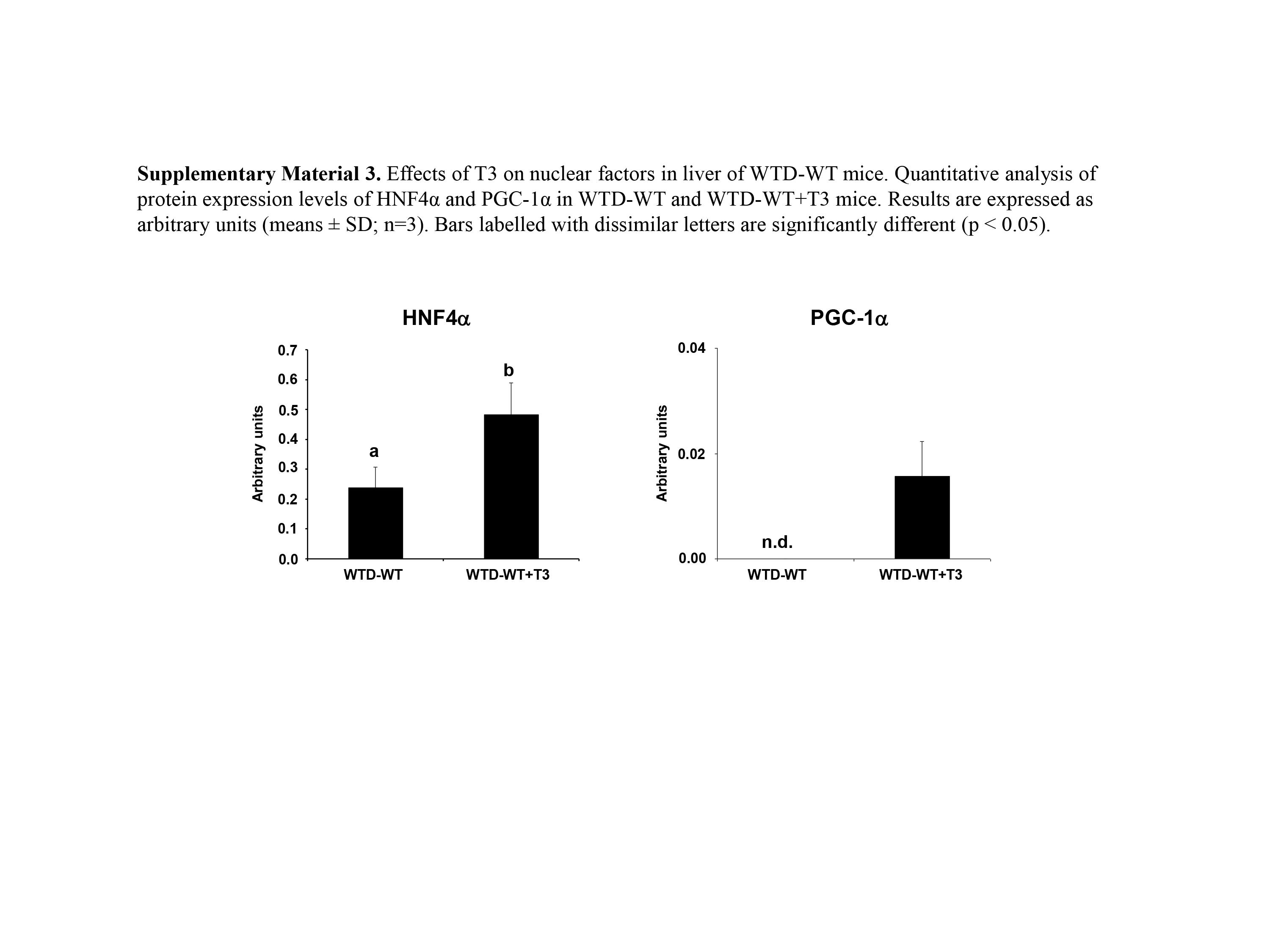

Supplement: Supplementary file 3 [file Image2.tif]
